# Longitudinal Analysis of Oral Potentially Malignant Disorder Conversion to Malignancy

**DOI:** 10.1002/lary.70199

**Published:** 2025-10-21

**Authors:** Benjamin Palatnik, Lindsey Mortensen, Aleksandr Palatnik, Beverly R. Wuertz, Frank G. Ondrey

**Affiliations:** ^1^ University of Minnesota Medical School‐Twin Cities Minneapolis Minnesota USA; ^2^ Itec Engineering LLC Pewaukee Wisconsin USA; ^3^ Department of Otolaryngology University of Minnesota Minneapolis Minnesota USA

**Keywords:** epidemiological studies, head and neck, oral cavity

## Abstract

**Objectives:**

Oral potentially malignant disorders (OPMDs) are local and systemic conditions that can result in oral malignancies. We have had an OPMD surveillance program for 25 years covering over 30% of our state's population. More recent electronic health record search techniques have allowed us to analyze over 1500 patients in over a 10‐year period, approximately 20% of total referrals.

**Methods:**

Electronic health record (EHR) information was queried for 24 International Classification of Disease (ICD) 9 and 10 codes to generate a de‐identified data set. Charts within this data set were cross‐referenced against 79 ICD9/10 codes corresponding to oral and oropharyngeal cancers. The time course of conversion and frequencies of precancerous and cancerous lesions was assessed.

**Results:**

Of 4496 unique patients seen, 1535 patient records met inclusion criteria. Seventy two patients showed cancerous conversion (4.69%), with 55 (3.58%) before 5 years, and 17 (1.11%) after a 5‐year surveillance period. Leukoplakia and OLP patients constituted 34.7% of conversions, though other lesions/diseases of the tongue/oral mucosa showed conversion. Tongue cancers were the most represented carcinomas, followed by carcinomas of the oropharynx.

**Conclusion:**

We discovered overall rates of malignant conversion within the broad range reported in the world literature (0%–60%). Importantly, this living data set can be repeatedly queried over time to discover significant malignant conversions, potentially over decades. With significant conversions occurring after 5 years, OPMD conditions likely require longer‐term surveillance like colon, breast, or other cancers.

**Level of Evidence:**

3.

## 1 Introduction

There are expected to be close to 60,000 oropharyngeal malignancies in the US in 2025 [1]. Many of these malignancies are preceded by oral potentially malignant disorders (OPMDs) [2, 3]. A plethora of OPMDs have been described over the past 40 years, including leukoplakia (OL), oral lichen planus (OLP), oral lichenoid lesions, Fanconi Anemia, solid organ and bone marrow transplantation history, glossitis, submucosal fibrosis, and more [4, 5, 6]. Classically, tobacco and alcohol are the principal risk factors for leukoplakia [7]. Leukoplakia, which serves as a typical index condition, is present in 2% of the population, and approximately 5% of these lesions will progress to oral cavity carcinoma over a five‐year period [8]. Further, prior smoking‐associated aerodigestive cancers are assumed to create “field carcinogenesis,” which is also technically an OPMD.

Although a large volume of work exists examining the epidemiology and pathophysiology of malignant transformation in OL [5, 6, 9], the extension of this work to other OPMDs remains largely incomplete. Since many of the OPMDs are chronic and indolent, these high‐risk conditions may persist for decades and not be captured accurately by record systems, particularly when patients migrate between health care systems [10, 11]. General guidelines exist for surveillance of the oral cavity for cancer, but the complex matrix of signs, symptoms, and risks spanning greater than 50 ICD 10 codes makes condition‐specific surveillance highly challenging. Screening at the primary care level, or in dentistry, makes sense but is challenging. Silk et al. conducted a 2018 survey‐based study of Family Medicine program directors and found that only 59% of respondents indicated that training was given for adult oral lesions including leukoplakia, lichen planus, and mouth ulcers. Only 31% of programs provided more than 4 h of oral health education in 3 years of residency [12, 13]. Therefore, referral of unfamiliar oral conditions might be best served by a more comprehensive surveillance program. Examples of large comprehensive oral lesions clinics have existed at the University of California San Francisco (1970s) or in British Columbia, Canada (currently) [6, 14].

Twenty‐five years ago, the University of Minnesota collaboratively formed a partnership between the senior author, a head and neck surgeon, and the Oral Medicine Division of the Dental School to create this kind of comprehensive oral lesions clinic. Considerable support came from the State (UM Med School Dean's office) and the Federal Government (NCI and NIDCR) to help serve this unmet need. This joint effort in patient recall and education, practitioner education, translational research in screening/cancer prevention/clinical trials, and regional/national outreach created a comprehensive clinic that receives over a 1000 visits a year in multiple health care systems for identification and surveillance of individuals at elevated risk for oral cancer development. At the University, this constitutes a tertiary/quaternary referral practice with patients coming in high numbers from a five‐state area and a total of 27 states overall [15].

In the current study, we examined the long‐term malignant conversion in tertiary OPMD patients from more than 24 ICD 9 and ICD 10 diagnosis codes. This high throughput, low‐cost search of OPMD conditions spanned at least 11 years, although some patients were clinically followed for several years before the EPIC medical record was adopted. We present that data here to give insight into the propensity for malignant conversion of a variety of OPMDs in the context of a complex tertiary/quaternary active surveillance program.

## 2 Materials and Methods

### 2.1 Patient Identification

**FIGURE 1:**
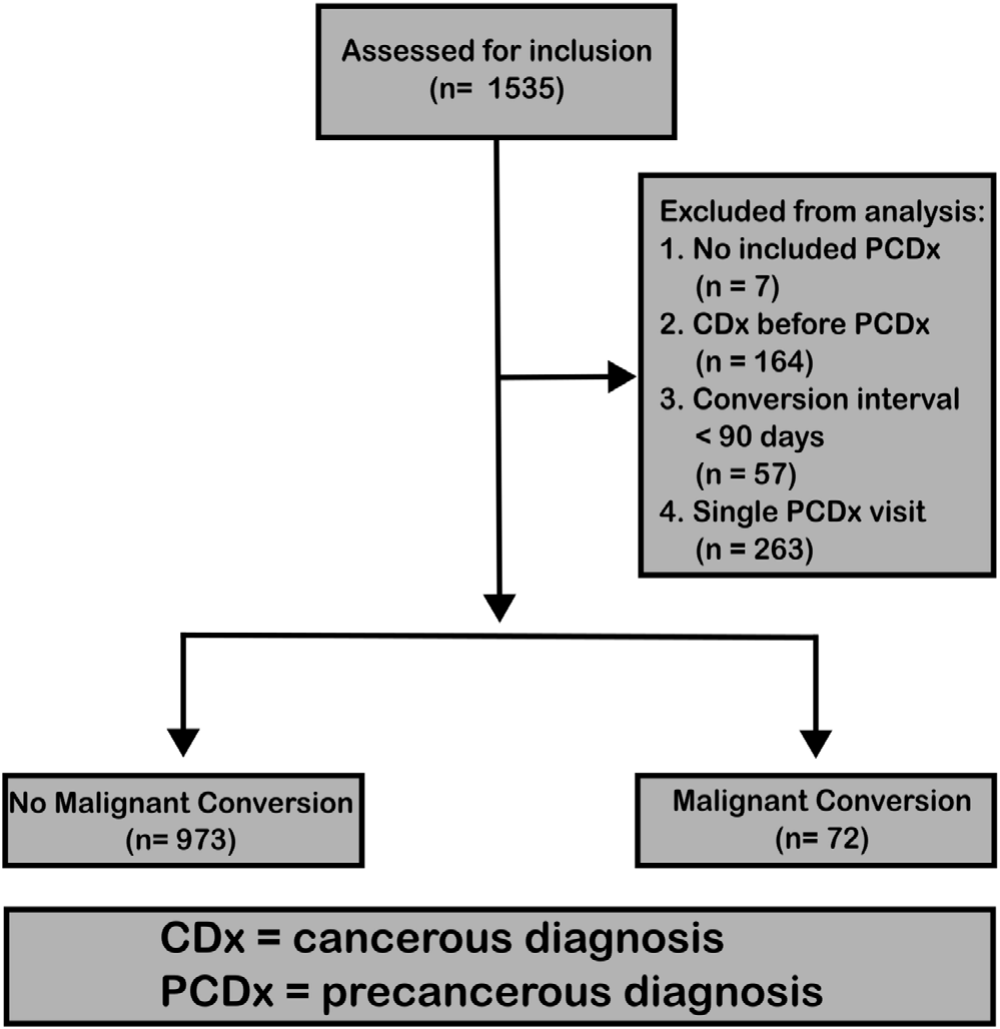
Patient inclusion schema. Schema describing the inclusion and identification process for patients with OPMDs who did and did not display malignant conversion.

To identify patients who were seen at the University of Minnesota Cancer Active Surveillance Program (CASP) for oral lesion screening, a collaboration was formed with the University of Minnesota's Clinical and Translational Science Institute (CTSI). A list of 24 International Classification of Diseases (ICD) 9 and 10 codes was developed corresponding to > 300 unique mucosal lesion descriptors associated with the potential development of oral cancer (Table S1). Extraction was performed as described by Dwyer and colleagues, with a final database lock date of January 13, 2023 [15]. The patient identification and conversion identification process is outlined in Figure 1.

### 2.2 Malignant Conversion

To assess the number of patients with malignant conversion of precancerous lesions, the subset of patients with the above pre‐cancerous ICD9/10 codes was filtered again with 79 ICD 9/10 codes corresponding to subsite descriptors for oral and oropharyngeal carcinoma in situ and cancer (Table S2). Patients with at least two visits related to OPMDs and a 90‐day minimum duration between initial OPMD and subsequent cancerous diagnoses were considered to have experienced a malignant conversion. A 90‐day minimum duration was chosen as lesion workups, treatment, and follow‐up for many conditions result in their resolution before 90 days. The development of an algorithm to identify conversion went through several iterations over the course of this study development. Improvements were made to increase the sensitivity of detection and rigorously define the appropriate time interval between OPMD and malignant lesion for inclusion as a conversion event. The final algorithm for malignant conversion was locked on February 10, 2025, and checked independently twice by two independent computer programming experts (A.P. and EP).

For tabulating conversion, patients with multiple diagnoses by various providers were assigned a primary OPMD upon tertiary referral. The primary diagnosis was determined by identifying either the most histopathologically specific diagnosis (e.g., OL, OLP, etc.) or the most disease‐specific diagnosis (e.g., Leukoplakia vs. stomatitis or glossitis). Carcinoma in Situ (CIS) was included in the cancer filter, as these lesions were often treated as early cancer and were histologic versus clinical diagnoses, like most other lesions.

Additionally, we report an unspecified lesion category, which is a more inclusive category for several diagnoses contributed by practitioners in the system, including but not limited to the senior author. These diagnoses could not be more specific for this type of study based on HIPAA regulations or ICD 9/10 limitations and could include a history of cancer, dysplasia pathologies, post radiation, second opinion consults for chronic inflammatory disorders, friction ridge, glossitis with lesion, etc.

### 2.3 Statistical Analysis

Descriptive statistics were used to assess variables in malignant conversion, including frequency and quantity, and time from precancerous diagnosis.

Chi‐squared tests were used to compare trends in categorical data including demographic data, diagnoses, etc. With unequal distributions, post hoc pairwise comparisons were utilized with a Bonferroni correction.

One sample, two‐tailed *t*‐tests were utilized to compare continuous data such as age at cancer diagnosis and intervals between precancerous and cancerous diagnoses with data reported in the literature, using published means for mu values. R (Vienna, Austria), GraphPad Prism Version 10 (Boston, MA), and Microsoft Excel were used for statistical analysis.

### 2.4 Ethical Considerations

The human subjects research reported here was reviewed and deemed exempt by the University of Minnesota Institutional Review Board (IRB # STUDY00016737) and the Clinical Protocol Review Committee of the MCC.

## 3 Results

**FIGURE 2:**
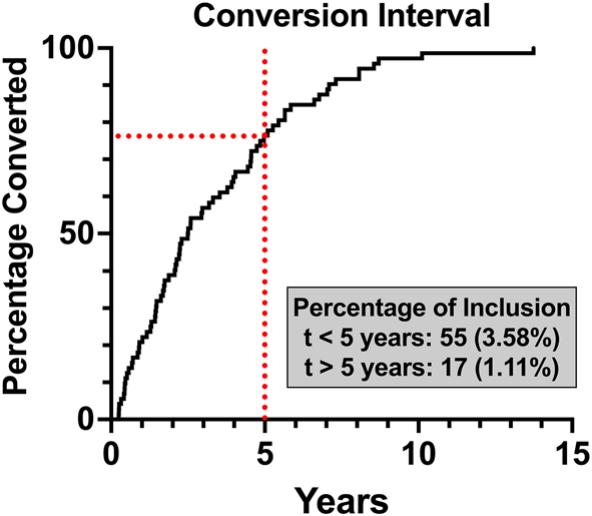
Malignant conversion over time. An inverted survival curve demonstrating the conversion interval for all patients who demonstrated malignant conversion in the inclusion cohort. The five‐year point is denoted with a red vertical dotted line. [Color figure can be viewed in the online issue, which is available at www.laryngoscope.com]

Of 4496 unique individuals seen over the study timespan, 1535 patients were identified with potentially precancerous oral lesions corresponding to 24 ICD 9 and 10 codes queried (i.e., not all possible ICD 9/10 codes for OPMDs were identified in this cohort). These patients were seen at 7371 visits over the study timespan and were often given at least two ICD codes from this list due to the refinement of diagnosis over multiple observations (mean = 1.96, SD = 1.40, Q3 = 2.0). Within this subgroup of 1535, 72 patients (4.69%) developed malignant disease. 55 individuals developed a malignancy within the first 5 years of OPMD diagnosis (3.58%). Among the ≤ 5‐year conversion cohort, the mean interval was 2.11 years (SD = 1.42, Q1 = 0.858, Q3 = 3.19). Seventeen patients converted after 5 or more years (1.11%), with the mean interval being 7.35 years (SD = 2.10, Q1 = 5.65, Q3 = 8.31, Max = 13.8). See Table 1 for lesion‐specific and overall conversion statistics and Figure 2 for a representation of conversion over time.

**TABLE 1:** Conversion data for all OPMDs studied in this work.

OPMD	Total CASP count (*n* = 1535)	Converted count (*n* = 72)	< 5‐year conversion	> 5‐year conversion	Total conversion frequency
Oral leukoplakia	149	20	17 (11.4%)	3 (2.01%)	13.42%
Oral lichen planus	384	23	13 (3.39%)	10 (2.60%)	5.99%
Glossitis	140	3	2 (1.43%)	1 (0.71%)	2.14%
Oral aphthae	84	2	2 (2.38%)	0	2.38%
Stomatitis	322	7	6 (1.86%)	1 (0.31%)	2.17%
Diseases of lips	17	2	1 (5.88%)	1 (5.88%)	11.76%
Personal history of other diseases of the digestive system	257	7	6 (4.96%)	1 (0.83%)	5.79%
Unspecified lesions	308	9	9 (2.92%)	0	2.92%
Total	1535	72	55 (3.58%)	17 (1.11%)	4.69%

Leukoplakia and OLP were the most common OPMDs to convert to cancer in this series (34.7% of total). The OPMD with the greatest conversion frequency was Oral Leukoplakia (13.4%, *n* = 20), followed by OLP (5.99%, *n* = 23) (Table 1). The cancers developed were heterogeneous, though the most common were cancers of the tongue (27.8%, *n* = 20) and unspecified locations within the oral cavity (20.1%, *n* = 15) (Table 2).

**TABLE 2:** Malignancy ICD codes identified among OPMD patients who demonstrated malignant conversion with associated frequencies.

ICD‐9/10 code	ICD code description	Count (*n* = 72)
C02.9, 141.8, 141.9	Malignant neoplasm of tongue, unspecified	20 (27.8%)
C06.9, 145.9	Malignant neoplasm of mouth, unspecified	15 (20.1%)
C01.0, 141.0	Malignant neoplasm of base of tongue	6 (8.33%)
C10.9, C14.0	Malignant neoplasm of oropharynx, unspecified	3 (4.17%)
C02.1, 141.2	Malignant neoplasm of border of tongue	3 (4.17%)
C06.0, 145.0	Malignant neoplasm of cheek	3 (0.86%)
C03.0	Malignant neoplasm of upper gum	2 (2.78%)
C04.1, C04.9	Malignant neoplasm of floor of mouth, unspecified	2 (2.78%)
C02.2	Malignant neoplasm of ventral surface of tongue	1 (1.34%)
C05.1	Malignant neoplasm of soft palate	1 (1.34%)
C05.9	Malignant neoplasm of palate, unspecified	1 (1.34%)
140.4	Malignant neoplasm of lower lip, inner aspect	1 (1.34%)
D04.30	Carcinoma in situ of skin of unspecified part of face	3 (4.17%)
230.0	Carcinoma in situ of lip, oral cavity, and pharynx	3 (4.17%)
D00.07	Carcinoma in situ of tongue	2 (2.78%)
D00.01	Carcinoma in situ of labial mucosa & vermilion border	1 (1.34%)
D09.90	Carcinoma in situ, unspecified	5 (6.94%)

We also performed an analysis of conversion by various demographic parameters. Not all race/ethnicity subgroups were represented in the conversion cohort, but those that were represented displayed frequencies consistent with overall statistics. Due to the small sample size of non‐White patients, rigorous analysis was underpowered. When comparing male and female patients, both groups had similar conversion frequencies (*p* = 0.470) (Table 3).

**TABLE 3:** Demography of patients who met overall inclusion criteria as well as those who demonstrated malignant conversion.

Race	Total CASP count (*n* = 1535)	Converted count (*n* = 72)	≤ 5‐year conversion	> 5‐year conversion	Total conversion frequency
White	1214	63	48 (3.95%)	15 (1.24%)	5.19%
Black/African American	124	4	3 (2.42%)	1 (0.81%)	3.23%
Asian	52	2	2 (3.85%)	0	3.85%
American Indian/Alaskan Native	12	—	—	—	—
Native Hawaiian/Pacific Islander	1	—	—	—	—
Mixed Race	6	—	—	—	—
Blank	126	3	2 (1.59%)	1 (0.79%)	2.38%
Sex					
Female	852	37	26 (3.05%)	11 (1.29%)	4.34%
Male	683	35	29 (4.25%)	6 (0.89%)	5.12%

To assess trends in age at conversion for OPMD patients, we compared experimental data against recent large‐scale epidemiological incidence rates. The patients in this study had a mean age at cancer diagnosis of 59.5 years (SD = 13.6, Q1 = 52.9, Q3 = 70.7, max = 81.6). This mean was slightly younger than the mean age at diagnosis of oral and oropharynx cancer reported in the SEER database (59.5 vs. 64, *p* = 0.0115) [16].

We next sought to compare the conversion intervals in our surveillance program against previously published frequencies in the literature, particularly the landmark work on oral leukoplakia conversion published by Silverman and colleagues [5]. Of the patients who developed cancer, the time interval from first OPMD to malignancy diagnosis was similar to those reported by the Silverman group in the first 10 years (this study vs. Silverman: 0.5–1 years: 16% vs. 9%, *p* = 0.152; 1–2 years: 12% vs. 24%, *p* > 0.999; 2–5 years: 27% vs. 20%, *p* > 0.111; 5–10 years: 15% vs. 22%, *p* > 0.999). Beyond 10 years, the patients in this study developed oral cancers at lower frequencies than those surveilled by Silverman et al. [5]. (10+ years 2% vs. 25%, *p* < 0.001).

It was of interest to determine whether OPMD patients who developed cancer were seen for cancer surveillance in accordance with guidelines we recently published and have been quoted at national meetings [17]. This evidence‐based protocol recommended surveillance visits every 3–6 months for OPMD patients within the first 5 years of diagnosis. Among those with a ≤ 5‐year interval between OPMD and cancer diagnoses, we observed a mean visit interval of 0.314 visits/month (SD = 0.361, Q1 = 0.0785, Q3 = 0.412, Max = 1.69), or nearly four visits/year, before cancer diagnosis, reflecting the protocol (*p* = 0.700). For patients whose cancer was diagnosed after 5 years, the mean visit interval was 0.157 visits/month (SD = 0.147, Q1 = 0.0413, Q3 = 0.274, Max = 0.476). Our protocol does not have specific guidelines for monitoring after 5 years. Predictably, patients were seen less frequently in the > 5‐year period (*p* = 0.0001).

## 4 Discussion

In this study, we were able to show the malignancy conversion rates of oral leukoplakia, oral lichen planus, and other conditions over a longer period than standard 5‐year cancer surveillance. The longstanding stability of the senior author's unique surveillance practice has currently allowed a 10‐year follow‐up for this project with the interrogation of 1.4 million medical records in our health care system to derive the patients analyzed here. The diagnoses are generally derived by referral practitioners and/or the senior author, who all have extensive oral lesion experience. Therefore, a greater diagnostic accuracy may exist compared to insurance record studies. A unique feature of this study is its cross‐sectional nature, analyzing a spectrum of ICD system‐classified lesions that have cancer conversion potential.

Our principal significant finding is that for large referral practices, there is considerable cancer conversion the longer the condition exists, so patients with these lesions and conditions likely need surveillance for long periods, perhaps even decades. Further, lesion removals do not treat the conditions and cancer preventive agents for these conditions do not exist [18, 19, 20]. The surveillance population analyzed in this study constitutes a longstanding tertiary/quaternary referral practice which regularly draws patients from a five‐state catchment in the Midwest and, to a lesser extent, nationally from at least 27 states. Because of the complex and collaborative nature of this surveillance clinic, many of these patients are referred due to recalcitrant, vexing, treatment‐resistant lesions or complex past medical histories like organ or bone marrow transplantation, Fanconi anemia, etc. Surveillance programs like ours are very rare but can serve a critical function for patients with these conditions. From the 72 individuals (4.69% of OPMD patients) who developed cancer, nearly 25% (1.11% of the total OPMD study population) developed cancer after 5 years of surveillance. Rates of oral cancer development were similar or slightly elevated in the < 5‐year conversion cohort relative to previous studies that likely were less tertiary [21, 22, 23].

Past studies have typically focused on oral leukoplakia (OL) conditions, finding conversion rates ranging from 0.13% to 17.5% with a large variation of follow‐up duration [4, 5, 6, 8, 9, 23]. With the reduction in tobacco use in the United States, overall OL conversion has been adjusted to 3.3%, though variability exists based on the degree of dysplasia (ranging from 2.2% with no dysplasia to 32.2% with severe dysplasia) [7]. In our cohort, conversion rates are consistent with both early and modern estimates in the context of severe dysplasia (13.42% overall, 11.4% < 5 years, 2.01% > 5 years), though one limitation of our dataset is the absence of explicit dysplasia stratification in the ICD 9 or 10 coding systems. In the future, natural language processing of EMR pathology can be employed to enhance specificity in this type of record interrogation [24, 25, 26, 27]. Nonetheless, construction databases that are specific for OPMD conditions that transcend years to decades are ostensibly nonexistent or financially impossible to fund, making this type of data collection a living database that remains contemporary with EMR systems like EPIC.

Oral lichenoid lesions and OLP have seen great variation in description and consideration as premalignant lesions over decades [28, 29, 30]. The pathology interpretation is difficult and ambiguous as well [31, 32]. For this reason, oral pathologists ask for extensive history and clinical correlation to key them in for histological diagnosis. Conversely, Wickham's striae on exam are pathognomonic, making non‐erosive OLP primarily a clinical diagnosis which can be augmented or substituted with histopathology for ICD coding purposes. Therefore, our approach takes into account the more accurate clinical diagnostic criteria that may be augmented with pathology. For malignant conversion, one large analysis found malignant conversion rates from 1.09% to 1.14% with an oral lichenoid lesions transformation rate of 3.2% in non‐dysplastic lichenoid lesions [33]. A 2020 meta‐analysis of 82 OLP and oral lichenoid lesions studies yielded average transformation rates of 1.14% and 1.88%, respectively but contained individual studies with conversion rates as high as 3.5% [3]. The elevated malignant conversion of OLP patients in our study (5.99% overall, 3.398% ≤ 5 years, 2.6% > 5 years) is similar to recent updates on transformation rates in dysplastic OLP (5.13%) [34]. This is due to several factors. Firstly, it reflects the recalcitrant nature of difficult OLP lesion patients in our tertiary referral practice, as described above. Secondly, patients coming in for second opinion to our referral clinic with well‐controlled disease or second opinions about their condition were not included in this study cohort (2 visit minimum for inclusion). Thirdly, patients with verrucous leukoplakia often have lichenoid features in their pathology and were likely classified as OLP. Further, with previous literature identifying recalcitrant disease in approximately 40% of OLP lesions, this would suggest an overall OLP conversion frequency of minimally 2.4% after accounting for single visit patients [35, 36, 37].

In this study, we included a cross section of OPMD conditions that have yet to be interrogated for their malignant potential. We report small conversion percentages for most of these conditions. As an example, glossitis demonstrated a 2.14% overall conversion rate. Tota et al. reported similar glossitis conversion statistics in a recent SEER database study, identifying glossitis histology in up to 3.9% of oral cancers [38]. “Diseases of lips” yielded a relatively high conversion frequency in this study, though this may be due to a low overall representation of this lesion type in our CASP. It is also fortunate that the majority of these less studied OPMDs demonstrated conversion within the first 5 years, making current screening guidelines valuable in those cases.

The patients in the “unspecified group” represent a number of diagnoses that are not as specific. The significant number of malignant conversions from this “unspecified group” (2.92%, Table 1) demonstrates one limitation of our automated database extraction process. This is expected because several conditions cannot be completely described with ICD 9/10 coding when manual chart reviews or highly specific expensive databases are not part of the query. This also could be resolved with more complex natural language processing [24, 25, 26, 27]. For other types of studies for more common public health conditions, large epidemiologic efforts in the multiple health systems are often supported by NIH, PCORI, or similar funding. Oral cancer surveillance in the field of OPMD would greatly benefit from such larger efforts due to the stagnation of cure rates, long durations of surveillance (*t* > 10 years), and ongoing education of health care practitioners and OPMD patients. The senior author's own practice has discovered new cancers in his patients up to 53 years after the original diagnosis of lichenoid conditions, for example (personal communication). Screening timelines should mirror the order of mammogram or colonoscopy recommendations—perhaps decades. Highly tertiary patient populations such as the one studied here provide great insight into longstanding conversions and represent a valuable observation group for the advancement of screening protocols.

## 5 Conclusion

Here we present an automated database longitudinal extraction of OPMD patients to highlight the malignant potential of these conditions, particularly in patients with complex lesions requiring tertiary referral. We demonstrate malignant conversion in the most frequently observed OPMDs, which is consistent with previous literature while highlighting the substantial quantity of malignant conversions occurring more than 5 years after initial diagnoses. With this in mind, we feel that patients should be seen for greater than 5 years and education on symptoms of malignant conversion be taught to potentially minimize negative impacts on disease progression and survival [39, 40, 41].

## Disclosure

The authors have nothing to report.

## Conflicts of Interest

The authors declare no conflicts of interest.

## Supporting information


**Table S1:** OPMD ICD 9/10 codes.
A complete list of all ICD 9 and 10 codes associated with OPMDs included in this study. The appropriate ICD code description is provided.

**Table S2:** Malignancy ICD 9/10 codes.
A complete list of all ICD 9 and 10 codes associated with malignancies included in this study. The appropriate ICD code description is provided.

## Data Availability

The data that support the findings of this study are available on request from the corresponding author. The data are not publicly available due to privacy or ethical restrictions.
