# Supplementary material for: Longitudinal Analysis of Oral Potentially Malignant Disorder Conversion to Malignancy
Source: Laryngoscope. 2025 Oct 21;136(4):1755–61. doi: 10.1002/lary.70199 (PMC12993112; doi:10.1002/lary.70199)
Supplement: Supplementary file 1 — Table S1: OPMD ICD 9/10 codes. A complete list of all ICD 9 and 10 codes associated with OPMDs included in this study. The appropriate ICD code description is provided. [file LARY-136-1755-s002.docx]

Supplementary Table 1

|  | **ICD Code** | **ICD Code Description** |
| --- | --- | --- |
| **ICD-9** | 528.0 | Stomatitis and mucositis, unspecified |
|  | 528.2 | Oral aphthae |
|  | 528.5 | Diseases of lips |
|  | 528.6 | Leukoplakia of oral mucosa, including tongue |
|  | 528.9 | Other and unspecified diseases of the oral soft tissues |
|  | 697.0 | Lichen planus |
| **ICD-10** | K12.1 | Other forms of stomatitis |
|  | K13.21 | Leukoplakia of oral mucosa, including tongue |
|  | K13.4 | Granuloma and granuloma-like lesions of oral mucosa |
|  | K13.5 | Oral submucous fibrosis |
|  | K13.70 | Unspecified lesions of oral mucosa |
|  | K13.79 | Other lesions of oral mucosa |
|  | K14.0 | Glossitis |
|  | K14.8 | Other diseases of tongue |
|  | K14.9 | Disease of tongue, unspecified |
|  | L43.0 | Hypertrophic lichen planus |
|  | L43.1 | Bullous lichen planus |
|  | L43.2 | Lichenoid drug reaction |
|  | L43.3 | Subacute (active) lichen planus |
|  | L43.8 | Other lichen planus |
|  | L43.9 | Lichen planus, unspecified |
|  | Z83.0 | Family history of human immunodeficiency virus [HIV] disease |
|  | Z83.79 | Family history of other diseases of the digestive system |
|  | Z87.19 | Personal history of other diseases of the digestive system |
