# Supplementary material for: Longitudinal Analysis of Oral Potentially Malignant Disorder Conversion to Malignancy
Source: Laryngoscope. 2025 Oct 21;136(4):1755–61. doi: 10.1002/lary.70199 (PMC12993112; doi:10.1002/lary.70199)
Supplement: Supplementary file 2 — Table S2: Malignancy ICD 9/10 codes. A complete list of all ICD 9 and 10 codes associated with malignancies included in this study. The appropriate ICD code description is provided. [file LARY-136-1755-s001.docx]

Supplementary Table 2

|  | ICD Code | **ICD Code Description** |
| --- | --- | --- |
| **ICD-9** | 230.0 | Carcinoma in situ of lip, oral cavity, and pharynx |
|  | 231.0 | Carcinoma in situ of larynx |
|  | 232.0 | Carcinoma in situ of skin of lip |
|  | 140.3 | Malignant neoplasm of upper lip, inner aspect |
|  | 140.4 | Malignant neoplasm of lower lip, inner aspect |
|  | 140.5 | Malignant neoplasm of lip, unspecified, inner aspect |
|  | 141.0 | Malignant neoplasm of base of tongue |
|  | 141.1 | Malignant neoplasm of dorsal surface of tongue |
|  | 141.2 | Malignant neoplasm of tip and lateral border of tongue |
|  | 141.3 | Malignant neoplasm of ventral surface of tongue |
|  | 141.4 | Malignant neoplasm of anterior two-thirds of tongue, part unspecified |
|  | 141.5 | Malignant neoplasm of junctional zone of tongue |
|  | 141.6 | Malignant neoplasm of lingual tonsil |
|  | 141.8 | Malignant neoplasm of other sites of tongue |
|  | 141.9 | Malignant neoplasm of tongue, unspecified |
|  | 143.0 | Malignant neoplasm of upper gum |
|  | 143.1 | Malignant neoplasm of lower gum |
|  | 143.8 | Malignant neoplasm of other sites of gum |
|  | 143.9 | Malignant neoplasm of gum, unspecified |
|  | 144.0 | Malignant neoplasm of anterior portion of floor of mouth |
|  | 144.1 | Malignant neoplasm of lateral portion of floor of mouth |
|  | 144.8 | Malignant neoplasm of other sites of floor of mouth |
|  | 144.9 | Malignant neoplasm of floor of mouth, part unspecified |
|  | 145.0 | Malignant neoplasm of cheek mucosa |
|  | 145.1 | Malignant neoplasm of vestibule of mouth |
|  | 145.2 | Malignant neoplasm of hard palate |
|  | 145.3 | Malignant neoplasm of soft palate |
|  | 145.4 | Malignant neoplasm of uvula |
|  | 145.5 | Malignant neoplasm of palate, unspecified |
|  | 145.6 | Malignant neoplasm of retromolar area |
|  | 145.8 | Malignant neoplasm of other specified parts of mouth |
|  | 145.9 | Malignant neoplasm of mouth, unspecified |
|  | 146.0 | Malignant neoplasm of tonsil |
|  | 146.1 | Malignant neoplasm of tonsillar fossa |
|  | 146.2 | Malignant neoplasm of tonsillar pillars (anterior) (posterior) |
|  | 146.5 | Malignant neoplasm of junctional region of oropharynx |
|  | 146.6 | Malignant neoplasm of lateral wall of oropharynx |
|  | 146.7 | Malignant neoplasm of posterior wall of oropharynx |
|  | 146.8 | Malignant neoplasm of other specified sites of oropharynx |
|  | 146.9 | Malignant neoplasm of oropharynx, unspecified site |
|  | 149.1 | Malignant neoplasm of waldeyer's ring |
| **ICD-10** | C01 | Malignant neoplasm of base of tongue |
|  | C02.0 | Malignant neoplasm of dorsal surface of tongue |
|  | C02.1 | Malignant neoplasm of border of tongue |
|  | C02.2 | Malignant neoplasm of ventral surface of tongue |
|  | C02.3 | Malignant neoplasm of anterior two-thirds of tongue, part unspecified |
|  | C02.4 | Malignant neoplasm of lingual tonsil |
|  | C02.8 | Malignant neoplasm of overlapping sites of tongue |
|  | C02.9 | Malignant neoplasm of tongue, unspecified |
|  | C03.0 | Malignant neoplasm of upper gum |
|  | C03.1 | Malignant neoplasm of lower gum |
|  | C03.9 | Malignant neoplasm of gum, unspecified |
|  | C04.0 | Malignant neoplasm of anterior floor of mouth |
|  | C04.1 | Malignant neoplasm of lateral floor of mouth |
|  | C04.8 | Malignant neoplasm of overlapping sites of floor of mouth |
|  | C04.9 | Malignant neoplasm of floor of mouth, unspecified |
|  | C05.0 | Malignant neoplasm of hard palate |
|  | C05.1 | Malignant neoplasm of soft palate |
|  | C05.2 | Malignant neoplasm of uvula |
|  | C05.8 | Malignant neoplasm of overlapping sites of palate |
|  | C05.9 | Malignant neoplasm of palate, unspecified |
|  | C06.0 | Malignant neoplasm of cheek mucosa |
|  | C06.1 | Malignant neoplasm of vestibule of mouth |
|  | C06.2 | Malignant neoplasm of retromolar area |
|  | C06.80 | Malignant neoplasm of overlapping sites of other and unspecified parts of mouth |
|  | C06.89 | Malignant neoplasm of overlapping sites of unspecified parts of mouth |
|  | C06.9 | Malignant neoplasm of mouth, unspecified |
|  | C09.0 | Malignant neoplasm of tonsillar fossa |
|  | C09.1 | Malignant neoplasm of tonsillar pillar (anterior) (posterior) |
|  | C09.8 | Malignant neoplasm of overlapping sites of tonsil |
|  | C09.9 | Malignant neoplasm of tonsil, unspecified |
|  | C10.2 | Malignant neoplasm of lateral wall of oropharynx |
|  | C10.3 | Malignant neoplasm of posterior wall of oropharynx |
|  | D00.00 | Carcinoma in situ of oral cavity, unspecified site |
|  | D00.01 | Carcinoma in situ of labial mucosa and vermilion border |
|  | D00.07 | Carcinoma in situ of tongue |
|  | D00.08 | Carcinoma in situ of pharynx |
|  | D02.0 | Carcinoma in situ of larynx |
|  | D04.30 | Carcinoma in situ of skin of unspecified part of face |
